# Epidemiological and etiological profile of spinal cord injury in Brazil: a systematic review and meta-analysis

**DOI:** 10.1186/s40621-026-00700-6

**Published:** 2026-09-22

**Authors:** Sofia Gallina Ferreira, Guilherme Coelho, Orcizo Francisco Silvestre, Amadeu Zattoni da Silva, Arlete Maria Valente Coimbra, Alberto Cliquet Júnior

**Affiliations:** Faculdade de Ciências Médicas, Universidade Estadual de Campinas, Ciência Aplicada à Qualificação Médica, Campinas, SP Brazil

**Keywords:** Spinal Cord Injuries, Epidemiology, Brazil, Etiology, Health Promotion, Meta-Analysis as Topic

## Abstract

**Objectives:**

Spinal Cord Injury (SCI) severely impacts quality of life, yet consistent epidemiological data in Brazil are scarce. This study aimed to delineate the etiological profile of SCI in the Brazilian population to support public health strategies.

**Design:**

We conducted a systematic review and meta-analysis of proportions. Searches were performed in Medline, LILACS, and Embase. From the selected articles, 14 were included in the qualitative synthesis and 9 in the meta-analysis. The risk of bias was assessed using the JBI tool.

**Results:**

The meta-analysis with 8 studies revealed that the main etiologies of SCI were motor vehicle accidents (37.3%; 95% CI: 23.9–51.7), followed by falls (22.7%; 95% CI:16.2–29.9), violence including firearm injuries and physical assault (19.4%; 95% CI: 14.5–24.8), and diving into shallow water (10.9%; 95% CI: 5.1–18.3). Regarding the neurological level, the meta-analysis comprising 6 studies showed a pooled proportion of cervical injury of 37.4% (95% CI, 27.0-48.4). A male predominance was observed. We identified high heterogeneity (I² ranging from 76% to 95%) across all analyses, significant methodological weaknesses (e.g., hospital-based convenience samples), and evidence of publication bias for falls and violence.

**Conclusions:**

The etiological profile of SCI in Brazil reveals motor vehicle accidents as the leading cause, followed by falls and violence; and the most commonly affected neurological level is the cervical region. However, high heterogeneity and the risk of bias in primary studies demand a cautious interpretation of these findings. There is an urgent need for methodologically robust population studies to discover reliable data that can assist in effective public policies for the prevention of spinal cord injuries.

**Supplementary Information:**

The online version contains supplementary material available at https://doi.org/10.1186/s40621-026-00700-6.

## Introduction

 Spinal Cord Injury (SCI) is a high-impact neurological condition characterized by variable impairment of motor, sensory, visceral, and autonomic functions, depending on the injury’s severity and level [1]. The consequences of SCI transcend the physical impact, resulting in psychoaffective changes and requiring complex, costly care, imposing a significant burden on individuals, their families, and health systems [2].

Globally, SCI incidence varies significantly between developed and developing countries, with estimates ranging from 12.7 to 52.2 new cases per million inhabitants annually in different populations [3]. In Brazil, the estimated number of new cases ranges from 16 to 26 per million inhabitants per year [4]. However, these data are often limited and inconsistent, hindering a thorough and comparable epidemiological understanding [3, 4].

In Brazil, despite SCI being recognized as a highly complex and irreversible condition with high care needs and health costs [2, 5], there is a lack of appropriate services and limited preparation of health professionals for the comprehensive management of these patients [6]. This gap is particularly critical in Primary Health Care (PHC), where a lack of training can compromise health promotion and the prevention of secondary complications [6]. Educating PHC professionals is crucial for improving the longitudinal and specific care that is essential for individuals with SCI [6].

Accurate identification of SCI etiologies is imperative for designing preventive strategies and formulating effective public policies [1]. In Brazil, the main causes have historically included traffic accidents, firearm injuries, and falls [4]. The dynamics of these causes are influenced by sociodemographic and contextual factors, such as violence, urbanization, and the processes of demographic transition and population aging [7]. Although Brazil has legislation aimed at the inclusion of people with disabilities, such as the National Plan for the Rights of Persons with Disabilities – Living Without Limits [8] and the Quota Law for People with Disabilities [9], the effectiveness of these measures depends on up-to-date epidemiological knowledge that considers the country’s vast cultural, economic, and geographic heterogeneity.

Given the dispersion and variability in the findings of isolated observational studies on the etiologies of SCI in Brazil, a systematic synthesis of the available evidence through meta-analysis is essential. Meta-analysis allows for the quantitative combination of results from multiple studies, providing more robust prevalence estimates and assessing the consistency of findings across different populations and time periods [4]. This approach is crucial for overcoming the limitations of individual studies and providing a consolidated picture that can inform clinical decision-making and the prioritization of public health interventions.

Therefore, the aim of this study was to conduct a meta-analysis of the proportions of the leading causes of SCI in Brazil. Specifically, we sought to estimate the pooled prevalence of SCI attributed to traffic accidents, firearms, falls, and diving in shallow water, assess heterogeneity, and graphically illustrate the results, in order to provide an updated and comprehensive overview of the epidemiology of SCI causes in the Brazilian context.

## Methods

This systematic review and meta-analysis was registered with PROSPERO (CRD420251132610) and reported according to the Preferred Reporting Items for Systematic Reviews and Meta-Analyses (PRISMA) guidelines.

### Eligibility criteria

Observational studies (cross-sectional, cohort, and case-control) that investigated the epidemiology of SCI in Brazil were included. There were no restrictions on language or year of publication. We excluded reviews (narrative, systematic, and scoping), editorials, posters, letters to the editor, in vitro or animal studies, and studies that focused exclusively on vertebral trauma or other spinal disorders without confirmed involvement of the spinal cord.

### Search strategy

A comprehensive search strategy was developed and applied to the following electronic databases: MEDLINE (via PubMed), Embase, and LILACS (Latin American and Caribbean Literature on Health Sciences). The search was conducted in August 2025. Controlled terms (MeSH - Medical Subject Headings and DECS - Health Sciences Descriptors) related to SCI, epidemiology, and Brazil were combined using Boolean operators (AND, OR) to construct the search query.

The full search strategy for each database is available in the Supplemental Material (see File S1).

### Study selection and data extraction

Study selection was performed by two independent reviewers (R1 and R2). Initially, titles and abstracts were independently screened to identify potentially eligible studies. Subsequently, the full texts of these selected studies were retrieved and assessed for eligibility by R1 and R2 against the inclusion criteria and, at this time, articles that could not be accessed in full were excluded from the review. Any disagreements were resolved through discussion, and, if necessary, a third reviewer (R3) was consulted.

Data extraction was performed independently by R1 and R2 using a standardized form. The extracted data included: study information (authors, year of publication), population characteristics (age, sex), sample size, criteria used to define SCI and SCI etiology.

### Risk of bias assessment

The risk of bias in each included study was independently assessed by R1 and R2 using the Joanna Briggs Institute (JBI) Critical Appraisal Tool for Prevalence Studies. This tool assesses nine methodological domains (Q1–Q9) related to internal and external validity. The domains are: (Q1) appropriate sample frame, (Q2) appropriate recruitment/sampling, (Q3) adequate sample size, (Q4) detailed description of subjects and setting, (Q5) sufficient data analysis coverage, (Q6) valid methods for identifying the condition, (Q7) standardized and reliable measurement of the condition, (Q8) appropriate statistical analysis, and (Q9) adequate response rate. Each domain was rated as “yes” (low risk of bias), “no” (high risk of bias), “unclear” (unclear risk), or “not applicable.” The results of this assessment were summarized in a table. Discrepancies were resolved through discussion or consultation with R3.

### Data synthesis and statistical analysis

Data were qualitatively synthesized through a textual description and tabular presentation of study characteristics. For the meta-analysis, pooled proportions were estimated with a random-effects model (DerSimonian-Laird estimator) after the Freeman-Tukey double-arcsine transformation, which stabilizes the variance of proportions and accommodates estimates near 0 and 1.

Heterogeneity was assessed with the I² statistic and the between-study variance (τ²), and 95% prediction intervals were computed. I² values were interpreted as low (0–25%), moderate (25–75%) or high (> 75%).

Random-effects meta-regression was used to examine the year of data collection and the JBI risk-of-bias rating as potential moderators; geographic region was not modeled, since almost all studies were single-center and region could not be estimated with adequate precision. Small-study effects were examined with funnel plots and the Egger test. For the etiology outcome, a sensitivity analysis was performed by excluding the single study rated as high risk of bias. A second sensitivity analysis restricted the etiology and neurological-level pools to studies whose numerator and denominator could be confined to confirmed spinal cord injury (patients with a neurological deficit); studies enrolled under a broad spinal-trauma frame that did not report the mechanism or level of injury separately for deficit-positive patients were excluded from this analysis.

All statistical analyses were performed in Python 3.12.3 using NumPy 2.4.4, SciPy 1.17.1, pandas 3.0.2 and Matplotlib 3.10.8.

## Results

### Study selection process

The electronic database search yielded 163 records. After the removal of 43 duplicates, 120 articles were screened by title and abstract, of which 97 were excluded. Reasons for exclusion were: studies focusing on secondary complications of SCI, instrument validation, quality of life assessment or any different characteristic that was defined in our presented eligibility criteria.

Consequently, 23 articles [10–32] were retrieved for full-text evaluation and included in the qualitative synthesis. Of these, 2 studies [10, 11] could not be located in their entirety because they are stored in physical libraries and have been removed from the review at this time. In the end, 21 studies were eligible and had their full-text read [12–32].

During the evaluation, 7 articles were removed from the revision. Three articles had the definition of SCI only by self-report without clinical or imaging confirmation [12–14], two studies had their sample size compared to that of another article and the article with the larger sample size was retained [15, 16]. One reference the sample consists of hospital data from the inpatient system and not access to medical records which reduces the accuracy of the data obtained [17] and one study only analyzed patients who underwent surgical treatment, which is a limiting factor of the sample [18].

**Fig. 1:**
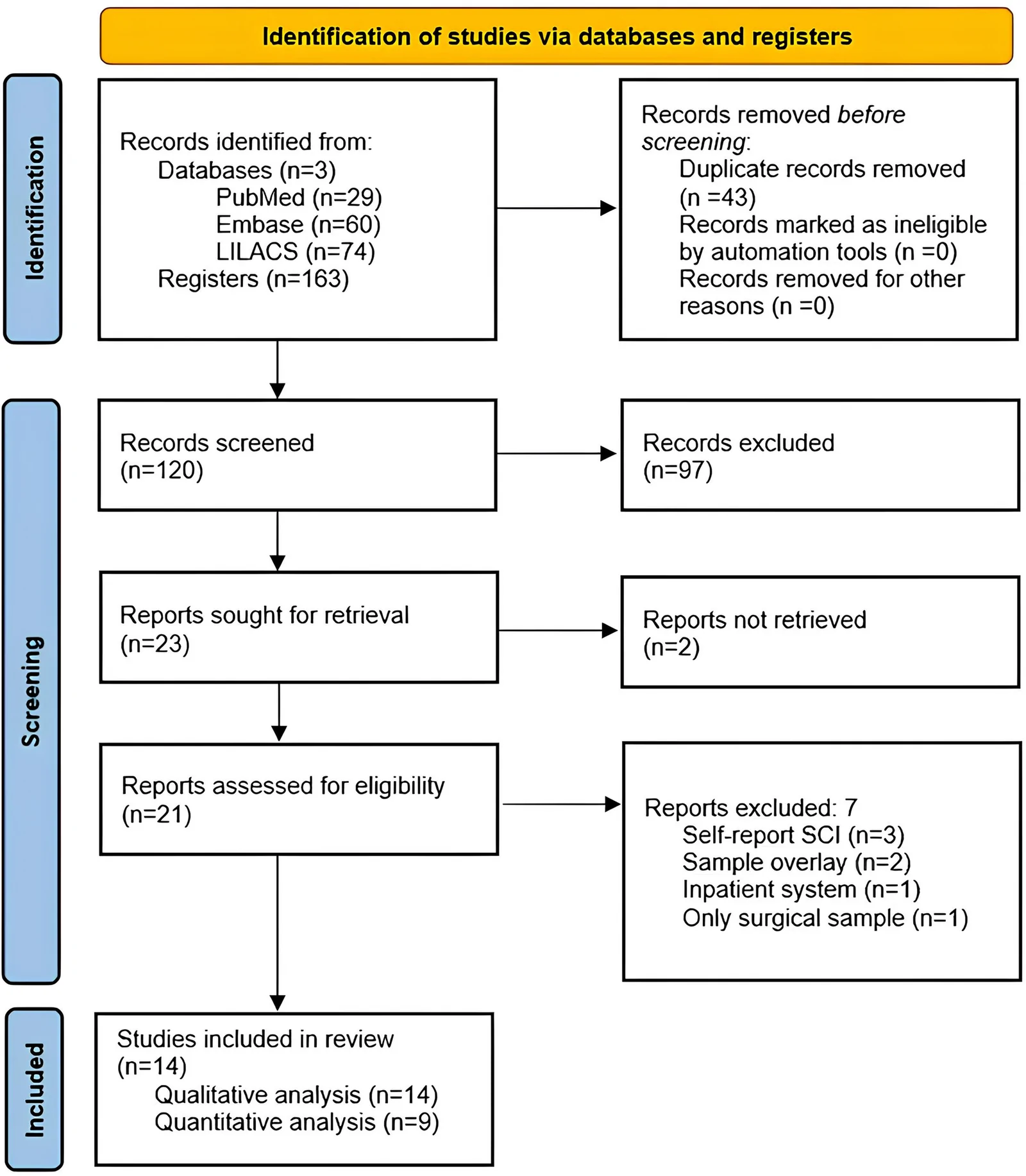
PRISMA flowchart of the selection process of studies included in the quantitative meta-analysis. Adapted from the PRISMA 2020 flow diagram template for new systematic reviews with searches of databases and registers only.

In the end, 14 studies were included in the review [19–32], of which 9 contributed to the quantitative synthesis (Fig. 1) [21–32].

## Characteristics of included studies

The 14 studies, characteristics, population definitions and methodological features were summarized in Table 1. Of the 14 studies included in the qualitative synthesis 12 had first authors who were physicians, one a nurse, and one a physiotherapist. All were published in relevant scientific journals in the field. The study sample sizes ranged from 41 to 808 patient records, with most studies conducted in the 2000s, the oldest dating from 1990 and the most recent from 2023 [19–32].

Regarding the data collection setting, 9 studies used hospitalized patients, while 5 included rehabilitation center attendees.

Two studies were included only in the qualitative assessment of the epidemiological profile because they presented a restricted sample of pediatric patients [19] and another of elderly patients [20].

Most studies were conducted in the Southeast region of Brazil (six in the state of São Paulo) which concentrates research and rehabilitation centers [19, 20, 22, 25, 26, 30]. Other locations included Goiânia [32], Belém [31], Porto Alegre [27–29], Curitiba [21], Palmas [23] and one study covering seven capitals of Brazil [24].

Data on education, marital status, salary/income, and employment were limited, with few studies addressing these aspects.

Regarding the epidemiological profile, all 14 included studies provided data on sex and age, and 13 reported SCI etiology. A male predominance was observed in all studies, with proportions ranging from 56% to 85% [19–32]. The most frequently affected age group was young adults (21–40 years); more recent studies (post-2015) have indicated an increase in the average age of patients with injuries associated with falls, possibly reflecting population aging [20].

The main etiologies of SCI identified were traffic accidents, firearm injuries, falls, and shallow-water diving. In older studies (1992 to 2010), firearm injuries accounted for a significant percentage [19, 24, 30]. In more recent studies (post-2015), traffic accidents and falls constituted the majority of cases [20–23, 25–27, 32].

**Table 1:** Characteristics, population definitions and methodological features of the 14 studies included in the qualitative synthesis

Study (year)	Region (state)	Setting and design	Data collection period	Sample size, *N* (effective SCI *N*)ᵃ	Population frame	Neurological deficit requiredᵇ	Non-traumatic SCI	Neurological classification	Predominant etiologyᶜ	JBI scoreᵈ	Contribution to meta-analysisᵉ
Araújo Júnior et al. (2021)	Curitiba (PR)	Three tertiary trauma hospitals; retrospective	2018	705 (36)	Broad spinal trauma	No	Excluded	Non-standardized / NR	Traffic accidents	5/9 (moderate)	Not pooled (broad frame)
Bellucci et al. (2015)	São Paulo (SP)	Rehabilitation centre; cross-sectional	2012	348	Confirmed traumatic SCI	Yes	Excluded	ASIA (full)	Traffic injury (38·5%)	9/9 (low)	Etiology; level
Castro et al. (2015)	Palmas (TO)	Tertiary public hospital; prospective	2011–2012	59	Broad spinal trauma (39% Frankel E)	No	Excluded	Frankel	Traffic / motorcycle accidents	6/9 (moderate)	Etiology
Costacurta et al. (2010)	São Paulo (SP)	Rehabilitation centre (AACD); retrospective	2002–2008	106	Pediatric SCI; 50·9% traumatic, 49·1% non-traumatic	Yes	Included (49·1%)	Paraplegia / quadriplegia	Gunshot (42·6% of traumatic)	5/9 (moderate)	Age-stratified (pediatric)
da Paz et al. (1992)	Seven capitals (multi-region)	36 public hospitals; cross-sectional point-prevalence survey	1988	108	Confirmed traumatic SCI	Yes	Excluded	Para-/tetraplegia ± deficit	Traffic accidents (42%)	6/9 (moderate)	Etiology
de Andrade Lopes et al. (2021)	São Paulo (SP)	Specialised SCI clinic (POLEM); retrospective	NR	113	SCI; 54% traumatic, 46% non-traumatic (mixed)	Yes	Included (46%)	NR	Traffic accidents (traumatic subgroup)	7/9 (low)	Etiology; level
de Melo-Neto et al. (2017)	São José do Rio Preto (SP)	Tertiary hospital; retrospective	2008–2012	62	SCI in older adults (clinical + CT/MRI)	Yes	Not specified / mixed	ASIA (full)	NR	8/9 (low)	Age-stratified (older adults)
de Paiva et al. (2023)	Campinas (SP)	Tertiary hospital; retrospective	NR	41	Confirmed post-traumatic SCI	Yes	Excluded	ASIA (AIS)	Traffic accidents (56·1%)	2/9 (high)	Etiology (sensitivity analysis)
Finger et al. (2021)	Porto Alegre (RS)	Tertiary neurosurgery hospital; retrospective	2013–2018	808 (131)	Broad spinal trauma; SCI incidence 16·7%	No	Excluded	Frankel	Falls from height (62·9%)	7/9 (low)	Not pooled (broad frame)
Freitas (1990)	Porto Alegre (RS)	Emergency hospital; retrospective, consecutive cases	1983–1985	100	Acute traumatic SCI	Yes	Excluded	Motor deficit (non-standardized)	Falls (54%); diving (37%)	6/9 (moderate)	Etiology; level
Frison et al. (2013)	Porto Alegre (RS)	Two public hospitals; retrospective	2003–2005	936 (142)	Broad spinal injury; SCI subset 10·7%	No	Excluded	Lesion level / deficit	Falls from high places (27·2%)	6/9 (moderate)	Etiology; level
Gaspar et al. (2003)	São Paulo (SP)	Rehabilitation centre (Lar Escola São Francisco); retrospective	1999–2001	171	SCI; traumatic + non-traumatic (mixed)	Yes	Included	Complete / incomplete	Firearm injury (30·1%)	6/9 (moderate)	Level
Souza Júnior et al. (2003)	Belém (PA)	Emergency hospital; prospective	2002–2003	250	Traumatic spinal injury (26·8% Frankel E)	No (26·8% Frankel E)	Excluded	Frankel	Falls (39·6%)	8/9 (low)	Etiology; level
Veronezi et al. (2018)	Goiânia (GO)	Emergency hospital; retrospective, cross-sectional	2013	265 (71)	Broad traumatic spinal trauma; ~50% without cord injury	No	Excluded	Complete / incomplete	Traffic accidents	4/9 (moderate)	Not pooled (broad frame)

SCI = spinal cord injury. JBI = Joanna Briggs Institute Critical Appraisal Checklist for Studies Reporting Prevalence Data. ASIA = American Spinal Injury Association Impairment Scale. AIS = Abbreviated Injury Scale. AACD = Associação de Assistência à Criança Deficiente. POLEM = Associação de Apoio às Pessoas com Lesão Medular. CT = computed tomography. MRI = magnetic resonance imaging. NR = not reported. PR = Paraná. SP = São Paulo. TO = Tocantins. RS = Rio Grande do Sul. PA = Pará. GO = Goiás

^a^ For studies whose sampling frame was broad spinal (vertebral) trauma, the effective SCI N (in parentheses) is the number of confirmed spinal cord injury cases within the sample; this value, rather than the total trauma sample, was used as the meta-analytic denominator

^b^ Indicates whether a confirmed neurological deficit was required for study inclusion

^c^ Reproduced as reported by each primary study; for broad-frame studies it refers to the whole spinal trauma sample rather than to the SCI subset

^d^ JBI risk of bias rated as low (score ≥ 7), moderate (4–6) or high (≤ 3); these cut-offs are an a priori decision of the review team, as the JBI tool does not specify validated thresholds

^e^ Castro and Frison contributed to pooled estimates only where counts could be restricted to confirmed SCI cases; Araújo Júnior, Finger and Veronezi were not pooled because such counts were not retrievable. Costacurta and de Melo-Neto were reserved for age-stratified analyses and excluded from the overall pooled estimates

Created by the authors with Python 3.12.3

### Risk of bias assessment

The risk of bias assessment revealed significant methodological limitations across multiple domains (Table 2). It was applied in the 14 studies in the review, comprising 9 studies intended for the quantitative synthesis, 2 studies reserved for age-stratified analyses (pediatric population [19] and older adults [20]) and 3 reserved only for qualitative synthesis [21, 27, 32] because the spinal cord injury sample from these studies could not be separated solely for those with confirmed spinal cord injury.

**Table 2:** Risk of Bias of included studies, according to the JBI Tool

	Study	Q1	Q2	Q3	Q4	Q5	Q6	Q7	Q8	Q9	Score	Risk
1	Andrade Lopes et al. (2021)	Y	Y	Y	Y	Y	Y	U	Y	U	7/9	Low
2	Araújo Júnior et al. (2021)	**N**	**Y**	**Y**	**Y**	**U**	**Y**	**N**	**Y**	**U**	**5/9**	**Moderate**
3	Bellucci et al. (2015)	**Y**	**Y**	**Y**	**Y**	**Y**	**Y**	**Y**	**Y**	**Y**	**9/9**	**Low**
4	Castro et al. (2015)	**U**	**Y**	**U**	**Y**	**Y**	**Y**	**Y**	**Y**	**U**	**6/9**	**Moderate**
5	Costacurta et al. (2010)	**Y**	**Y**	**Y**	**Y**	**U**	**U**	**N**	**Y**	**U**	**5/9**	**Moderate**
6	da Paz et al. (1992)	**Y**	**Y**	**Y**	**Y**	**Y**	**U**	**N**	**Y**	**U**	**6/9**	**Moderate**
7	de Melo-Neto et al. (2017)	**Y**	**Y**	**Y**	**Y**	**Y**	**Y**	**Y**	**Y**	**U**	**8/9**	**Low**
8	de Paiva et al. (2023)	**U**	**U**	**U**	**Y**	**N**	**N**	**N**	**Y**	**U**	**2/9**	**High**
9	Finger et al. (2021)	**N**	**Y**	**Y**	**Y**	**U**	**Y**	**Y**	**Y**	**Y**	**7/9**	**Low**
10	Freitas (1990)	**Y**	**Y**	**Y**	**Y**	**Y**	**U**	**N**	**Y**	**U**	**6/9**	**Moderate**
11	Frison et al. (2013)	**Y**	**Y**	**Y**	**Y**	**U**	**Y**	**N**	**Y**	**U**	**6/9**	**Moderate**
12	Gaspar et al. (2003)	**Y**	**Y**	**Y**	**Y**	**Y**	**U**	**N**	**Y**	**U**	**6/9**	**Moderate**
13	Souza Júnior et al. (2003)	**Y**	**Y**	**Y**	**Y**	**Y**	**Y**	**Y**	**Y**	**U**	**8/9**	**Low**
14	Veronezi et al. (2018)	**N**	**Y**	**Y**	**Y**	**U**	**N**	**N**	**Y**	**U**	**4/9**	**Moderate**

Y, yes; N, no; U, unclear

Created by the authors with Python 3.12.3

Of the 14 studies appraised, 5 showed low risk of bias, 8 moderate risk, and 1 high risk. Negative and unclear ratings were concentrated in items Q6 and Q7, which concern the validity and standardization of the condition measurement. This pattern reflects a systematic limitation of the set, which is the absence of a standardized neurological classification in most Brazilian studies. Only Bellucci [22] and de Melo-Neto [20] applied the ASIA scale in full, whereas Castro [23], Finger [27], and Souza Júnior [31] used the Frankel scale, which is convertible to ASIA; the remaining studies relied on non-standardized classifications (complete versus incomplete, plegia versus paresis, or “cord disruption”) or, in the case of de Paiva [26], on the Abbreviated Injury Scale, which is not a neurological scale.

The study by de Paiva [26], with a score of 2/9, was the only one classified as high risk of bias. It was kept in the etiology pool because its sampling frame is confirmed SCI, and a sensitivity analysis was performed by excluding it to test the robustness of the pooled estimates. Its severity data, reported with the Abbreviated Injury Scale rather than ASIA or Frankel, were not included in any analysis of neurological severity.

Conversely, the studies by Araújo Júnior [21], Finger [27] and Veronezi [32] reached scores of 5/9, 7/9 and 4/9 respectively even though item Q1 was not met, because the broad spinal-trauma sampling frame of these studies does not match the SCI focus of our review; these studies were therefore not pooled for etiology and for neurological level. The single-rater appraisal and the conventional, non-validated score cutoff (≥ 7 for low risk) are acknowledged limitations of this assessment.

### Meta-analysis results

Of the 14 studies retained after full-text assessment, 9 contributed to the quantitative synthesis; the two studies restricted to specific age groups [19, 20] and the 3 with problems in the sample [21, 27, 32] were not pooled in the overall estimates.

Because the included studies reported etiology and neurological level against heterogeneous denominators, each study was admitted to a given meta-analysis only when the corresponding counts referred to spinal cord injury cases rather than to the broader pool of vertebral trauma. This admission was decided on a study-by-study and outcome-by-outcome basis. Five studies enrolled patients under a broad spinal trauma frame: Araújo Júnior [21], Castro [23], Finger [27], Frison [29] and Veronezi [32]. Three of them (Araújo Júnior, Finger and Veronezi) reported etiology and neurological level only for the full trauma sample and were not pooled for either outcome. Frison [29] reported trauma mechanism and lesion level separately for the SCI subset (*n* = 142) and contributed to both pools. Castro [23], whose sample of 59 acute spinal-trauma patients included 39% with no neurological deficit at admission, contributed to the etiology pool, since trauma mechanism was reported for the entire sample under a homogeneous spinal-trauma frame. Souza Júnior [31], although enrolled as a traumatic spinal cord injury series, likewise included a minority of neurologically intact patients (26.8% Frankel E); together with Castro [23], it was therefore examined in the confirmed-SCI sensitivity analysis described below.

The meta-analysis of etiology comprised 8 studies (1,109 cases) [22–26, 28, 29, 31]; one [29] of these studies did not report shallow-water diving as a distinct category, so the diving estimate was based on 7 studies. The meta-analysis of neurological level comprised 6 studies (1,124 cases).

The proportion of traumatic versus non-traumatic injury was not meta-analyzed, because only three studies enrolled a genuinely mixed population (Costacurta [19], de Andrade Lopes [25], and Gaspar [30]); among these studies, the proportion of traumatic injury ranged from 50.9% to 75.5%, with non-traumatic causes dominated by tumors and infections.

Across the 8 studies included in the etiology analysis (1,109 cases), motor vehicle accidents were the most frequent mechanism, with a pooled proportion of 37.3% (95% CI, 23.9–51.7), followed by falls at 22.7% (95% CI, 16.2–29.9) and violence (combined firearm injuries and physical assault) at 19.4% (95% CI, 14.5–24.8). Shallow-water diving, estimated from the 7 studies that reported it as a distinct category, accounted for the smallest share, with a pooled proportion of 10.9% (95% CI, 5.1–18.3).

Study-specific proportions varied widely for every category: from 24.6% to 62.7% for motor vehicle accidents, from 14.8% to 54.0% for falls, from 3.4% to 28.2% for violence, and from 3.4% to 37.0% for shallow-water diving. The motor vehicle accident category was defined broadly, encompassing collisions involving motorcycles and automobiles as well as pedestrians struck by vehicles; this aggregation, adopted to maximize the number of poolable studies, does not capture the well-documented rise of motorcycle-related injury over the study period [12, 22].

Between-study heterogeneity was high for all four categories (I² = 76% for violence, 85% for falls, 89% for diving, and 95% for motor vehicle accidents; all *p* < 0.001). The 95% prediction intervals were correspondingly wide — 4.4% to 49.2% for falls, 1.3% to 86.1% for motor vehicle accidents, 5.8% to 38.2% for violence, and 3.2% to 41.6% for diving — indicating that the pooled proportions should be read as central tendencies rather than as precise national figures.

Random-effects meta-regression did not identify the year of data collection as a significant moderator for any category (all *p* > 0.10), indicating that the observed variability is not explained by calendar time alone; the JBI risk-of-bias rating was likewise non-significant for all categories except falls (*p* = 0.024), an isolated and weak signal that was not interpreted as substantive.

In the sensitivity analysis excluding the study rated as high risk of bias (de Paiva [26]), the pooled estimates were within 3.2% points of those obtained in the primary analysis (motor vehicle accidents, 34.9%; falls, 24.4%; violence, 19.6%; diving, 7.7%), indicating that the findings were robust to the exclusion of that study.

**Fig. 2:**
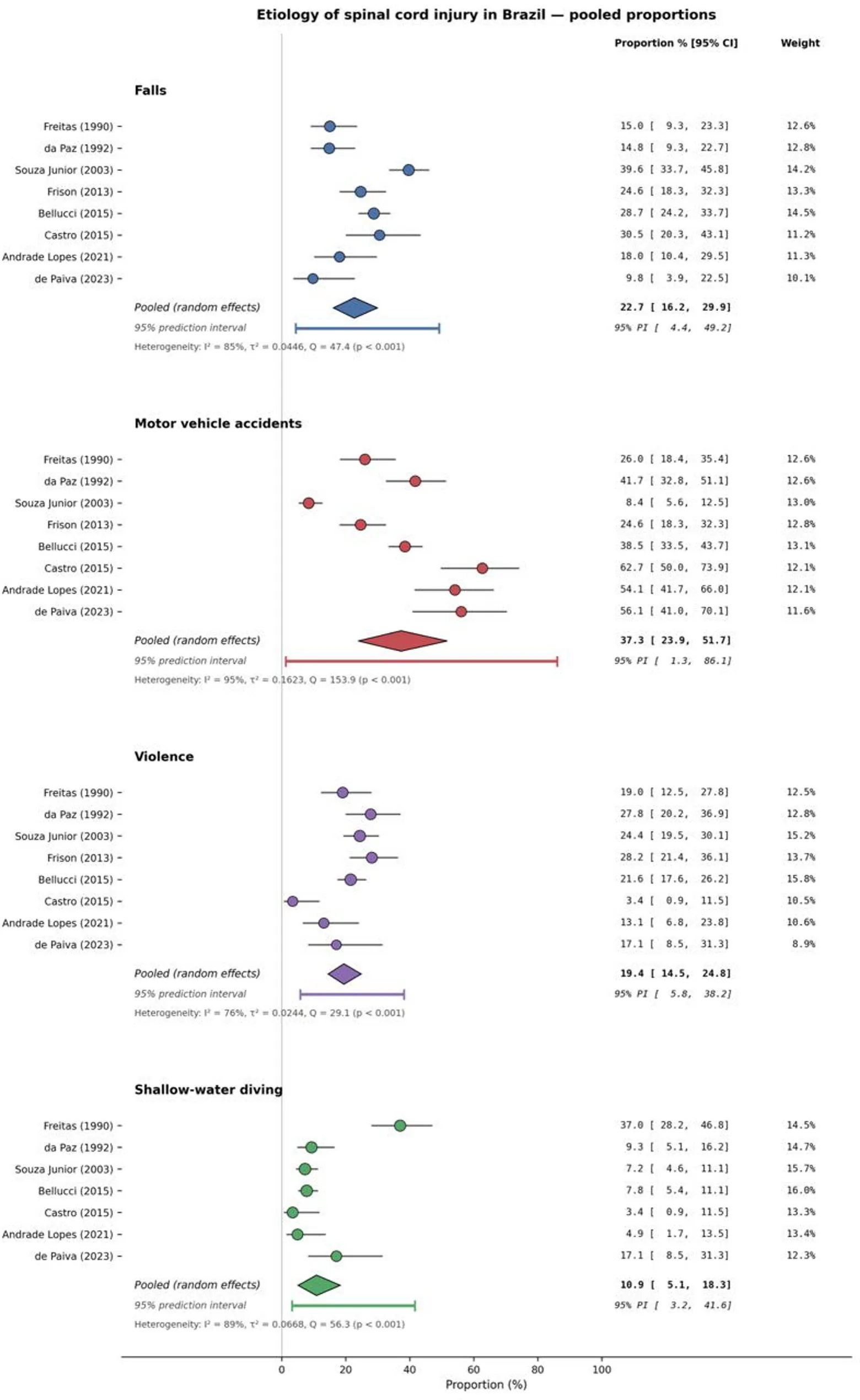
Forest plot of the pooled proportions for the four broad etiologic categories of spinal cord injury in Brazil. Squares represent study-specific proportions with 95% Wilson confidence intervals; square size is proportional to the study weight. Diamonds represent the random-effects pooled estimate, and the horizontal bars below each diamond represent the 95% prediction interval. The diving estimate is based on the 7 studies that reported diving as a distinct category. Created by the authors with Python 3.12.3

In a second sensitivity analysis, the etiology pool was restricted to studies with confirmed spinal cord injury. Two studies that enrolled patients under a broad spinal-trauma frame and included a substantial proportion of neurologically intact patients, Castro [23] (39.0% Frankel E) and Souza Júnior [31] (26.8% Frankel E), were excluded, because neither reported the mechanism of injury separately for deficit-positive patients. The restricted pool comprised 6 studies (800 cases; 5 studies for shallow-water diving). Pooled proportions were 38.8% (95% CI, 29.9–48.0) for motor vehicle accidents, 21.8% (17.8–26.2) for violence, 19.1% (13.4–25.4) for falls, and 13.7% (5.0–25.5) for shallow-water diving. All estimates were within 3.6% points of the primary analysis, every confidence interval overlapped its primary counterpart, and the etiologic ranking was preserved. Between-study heterogeneity decreased in the restricted pool (for example, I² for violence fell from 76% to 44%; for motor vehicle accidents, from 95% to 84%), indicating that part of the heterogeneity in the primary analysis was attributable to the inclusion of mixed spinal-trauma samples. When the same restriction was applied to the neurological-level outcome (excluding Souza Júnior [31]; Castro [23] did not contribute to that pool), the pooled proportion of cervical injury was 40.2% (95% CI, 28.7–52.2), compared with 37.4% (27.0–48.4) in the primary analysis. The etiologic and neurological-level profiles were therefore robust to the inclusion of broad spinal-trauma studies (Table S1, Figure S1, Fig. 2).

The neurological level was analyzed as a binary outcome (cervical versus non-cervical, with all levels below C7 combined). This dichotomy was adopted because it was the only partition reported consistently across the 6 included studies: the thoracolumbar junction (T11–L2) was isolated in only two studies, while the remaining four merged it into either the thoracic or the lumbar category, with classification criteria that could not be reconciled across studies. Pooling a three-level classification would therefore have combined non-equivalent definitions. The binary cervical-versus-non-cervical contrast avoids this definitional heterogeneity at the cost of anatomical detail.

**Fig. 3:**
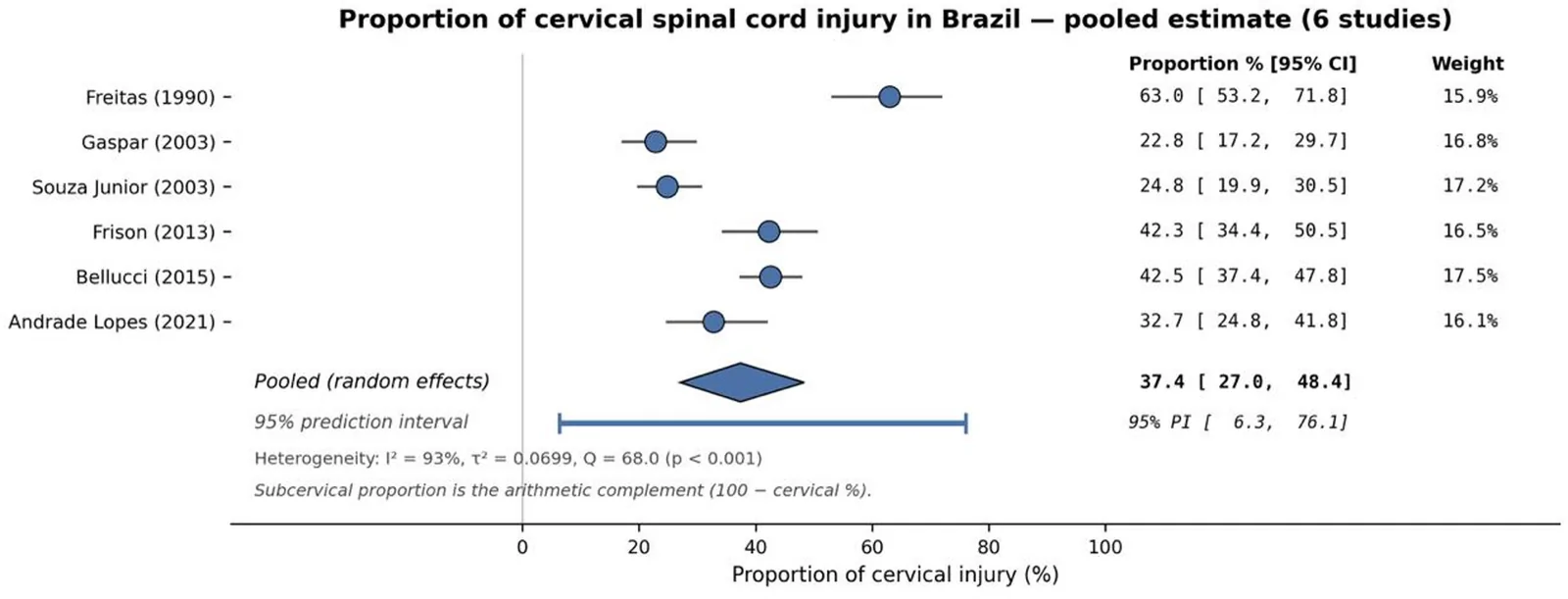
Forest plot of the pooled proportion of cervical spinal cord injury in Brazil. Squares represent study-specific proportions with 95% Wilson confidence intervals; square size is proportional to the study weight. The diamond represents the random-effects pooled estimate, and the horizontal bar below it represents the 95% prediction interval. The non-cervical proportion is the arithmetic complement of the cervical proportion. Created by the authors with Python 3.12.3

Among the 6 studies included (1,124 cases), the pooled proportion of cervical injury was 37.4% (95% CI, 27.0–48.4), as evidenced in Fig. 3. Study-specific cervical proportions ranged from 22.8% to 63.0%, with the highest value originating from a historical series (Freitas, 1990) that reported an unusually large share of diving-related injuries, a mechanism that predominantly affects the cervical spine.

Heterogeneity was high (I² = 93%; *p* < 0.001), and the 95% prediction interval for the cervical proportion was wide (6.3% to 76.1%); meta-regression did not identify the year of data collection as a significant moderator (*p* = 0.433), indicating that calendar time alone does not explain the observed variability. The Egger test did not indicate small-study effects (*p* = 0.129).

### Summary of qualitative findings and assessment of publication bias

Heterogeneity was high across all pooled outcomes and should be considered when interpreting the findings; the pooled proportions are therefore best read as broad summary estimates rather than as definitive national rates. This degree of heterogeneity was anticipated, given that the included studies span more than three decades (1983 to 2018) and cover four macro-regions of the country — with the Northeast represented only through a multicenter survey (da Paz [24]) — with differing case definitions and recruitment settings.

**Fig. 4:**
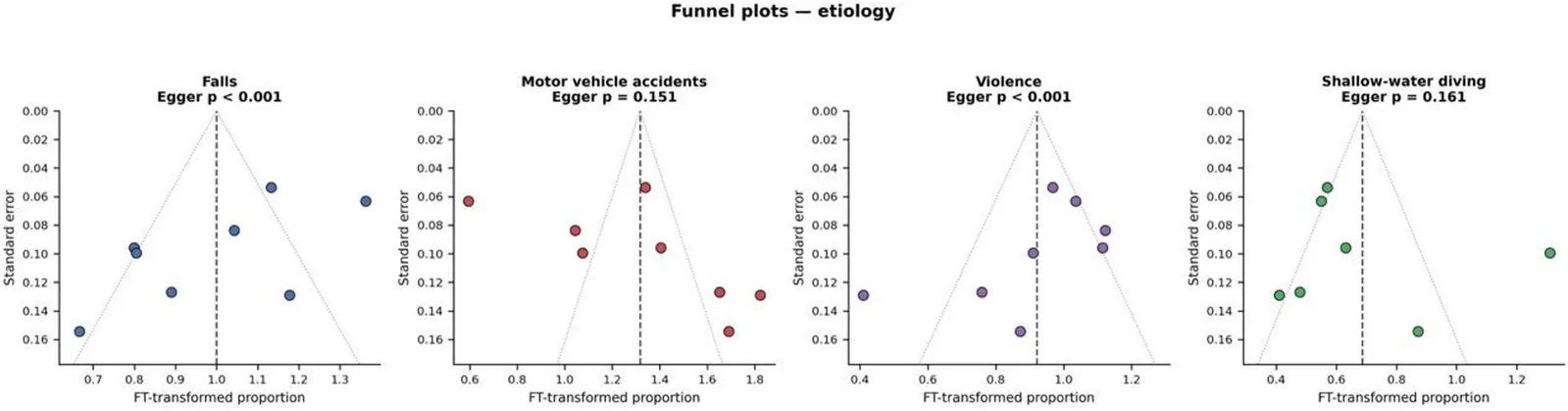
Funnel plots for the pooled proportion of each etiological category of spinal cord injury. Egger’s test p-values are displayed within each plot. Created by the authors with Python 3.12.3

The meta-regression results indicate that calendar time alone does not account for this variability; given that the JBI risk-of-bias rating also did not emerge as a significant moderator, the remaining heterogeneity likely reflects factors not modeled in this analysis, including regional and care-setting differences. As pre-specified in Methods, region and care setting could not be modeled because almost all studies were single-center, precluding adequate precision. Funnel plots are provided in Fig. 4.

The Egger test indicated possible small-study effects for falls and violence (*p* < 0.001 for both) but not for motor vehicle accidents (*p* = 0.151) or diving (*p* = 0.161); given the small number of studies, these tests have limited power and were interpreted only as a qualitative signal.

## Discussion

This meta-analysis of proportions represents an effort to delineate the epidemiological profile of the causes of SCI in Brazil, synthesizing the available evidence from 9 primary studies. Our findings reveal that motor vehicle accidents emerge as the main etiology (37.3%), followed by falls (22.7%), violence (19.4%), and diving into shallow water (10.9%); related to the neurological level, the most affected was the cervical region.

The identification of motor vehicle accidents as the most prevalent cause of SCI (37.3%) is consistent with the high burden of road traffic injuries in Brazil, particularly those involving motorcycles, as documented by the Institute for Applied Economic Research (IPEA) [33]. Violence, encompassing firearm injuries and physical assault, accounted for 19.4% of cases and demonstrates the persistence of urban violence as an important etiological factor in Brazil. Although some studies indicate a decrease compared to previous periods [4, 22], the recent expansion in the number of firearms in circulation in the country, according to data from the Atlas of Violence [34], suggests this etiology may continue to have a growing impact on SCI rates [34]. The predominance of males among SCI victims, reflected in all analyzed studies [19–32], also aligns with the profile of homicide and violence victims in Brazil, who are mostly young men [34, 35]. This phenomenon is multifaceted, involving social and cultural issues that shape risk behaviors and vulnerability [35]. As noted in the results, the statistical uncertainty of this estimate is highlighted by the wide confidence intervals in some primary studies, likely reflecting small sample sizes.

Falls were identified as the second most prevalent cause of SCI (22.7%). This is a notable finding that can reflect the changing demographic profile of the Brazilian population [20]. Traditionally, traffic accidents and violence were the predominant causes in Brazil, especially among young people, as documented both in older Brazilian series [24] and in national statistics [34]. However, Brazil’s demographic transition, with a progressive increase in the elderly population, can lead to a higher incidence of SCI in older age groups [20]. The forest plot for this outcome (Fig. 2) demonstrated substantial variability between studies, likely related to the different age groups included in the primary studies and the ongoing demographic transition, as falls are more prevalent in older populations [20]. These falls in the elderly are often associated with factors such as musculoskeletal weakness, decreased proprioception and balance, reduced vision, and polypharmacy [20], demanding special attention from health professionals, especially in Primary Health Care [6]. The implementation of tools such as the Pan American Health Organization (PAHO)’s Integrated Care for Older People (ICOPE) is fundamental for a broad assessment of this population’s functionality, allowing for the identification of risks and the prevention of adverse outcomes [36].

Traffic accidents (37.3%) represent the leading cause of SCI in this analysis. The high incidence of SCI related to motor vehicle accidents is consistent with data from the Instituto de Pesquisa Econômica Aplicada (IPEA), which indicates an increase in traffic deaths and substantial socioeconomic costs, mainly affecting the economically active population [33]. The prevalence of young males as the main victims of these accidents [34] highlights the need for traffic education campaigns, given that behavioral issues such as disobedience to rules and speeding still contribute significantly to these events [33]. The wide dispersion among study estimates seen in Fig. 2 is consistent with the regional, temporal and methodological heterogeneity discussed above.

In turn, diving into shallow water (10.9%) represents a specific cause, often associated with leisure and risk-taking behaviors. The relatively lower, yet still present, prevalence requires specific awareness campaigns, especially during holiday periods and at aquatic recreation sites. The variability in the forest plot for this cause (Fig. 2) may reflect geographic and seasonal differences, as these incidents are often linked to specific recreational activities.

A cross-cutting and high-impact finding in our meta-analysis was high heterogeneity (I² ranging from 76% for violence to 95% for motor vehicle accidents) observed in all proportion analyses. This heterogeneity cannot be attributed to chance and signals the existence of real differences among the primary studies. Such variations can be explained by multiple factors, many of which were identified in the methodological weaknesses of the studies, according to the risk of bias assessment using the JBI tool.

The primary studies included in this review had several limitations that contribute to the high heterogeneity and affect the external validity of our findings. First, most studies had a hospital-based design and used convenience samples, leading to a non-representative sample of the general population and likely underestimating cases that do not seek hospital care or that result in death before registration. This focus on acute trauma care leaves a gap in understanding longitudinal outcomes. Second, many studies lacked an explicit sampling method or a sample size calculation, and the wide variation in sample sizes (from 41 to 808 patients) complicates comparisons and generalizations. Third, incomplete insufficient detail on the validity of diagnostic instruments compromise the accuracy of the reported data. Finally, although not directly assessed by the JBI tool, most primary studies lacked control for potential confounders and relied on simple descriptive statistics, limiting a deeper understanding of factors associated with SCI.

A further consideration concerns population definition. Although most included studies restricted their samples to confirmed spinal cord injury, a few enrolled patients under a broader spinal-trauma frame that encompassed a minority of neurologically intact patients. A sensitivity analysis excluding the studies that could not be restricted to deficit-positive cases confirmed that the etiologic and neurological-level profiles were unchanged, although residual population heterogeneity cannot be entirely excluded.

The geographical concentration of studies in the Southeast region of Brazil, especially in São Paulo [4], despite the increase in scientific production in the country since the 1990s [37], weakens the representativeness of the data in a country of vast territorial heterogeneity. The scarcity of specific regional data limits the understanding of local particularities and the development of health policies adapted to the needs of each region.

Other important gaps identified, which limit the complete delineation of the epidemiological profile of the population with SCI, include the scarcity of data on education level, marital status, occupation, and income [19–32]. The limited available information suggests that individuals with SCI often have a lower level of education and face significant challenges in reintegrating into the labor market, with employment rates dropping drastically after the injury [1, 4]. This scenario contrasts with current legislation, such as Law No. 8.213/91 (Quota Law) [9], which aims to include people with disabilities in the workforce. The absence of multidisciplinary authorship in the analyzed articles [19–32] also reflects a gap in the holistic approach to SCI, which requires the collaboration of various health areas [6].

Finally, the predominance of data from studies conducted in the 2000s, with few more recent publications, indicates that the estimates may not reflect the current reality. Changes in health systems, public policies, and advances in the treatment and management of SCI may have altered the epidemiological profile, reinforcing the need for updated studies with more robust methodological designs.

In summary, this meta-analysis provided estimates of the causes of SCI in Brazil, highlighting motor vehicle accidents as the leading cause, the significant burden of falls and violence, and the persistence of shallow-water diving as a preventable etiology. However, the methodological limitations of the primary studies, the high heterogeneity, and the presence of publication bias for falls and violence demand caution in generalizing the results. These findings reinforce the need for investment in future research that employs more rigorous methodologies, population-based data representative of all Brazilian regions, and multidisciplinary approaches for a comprehensive understanding of the needs of individuals with SCI and for the development of more effective public policies.

## Conclusion

This systematic review and meta-analysis provide a synthesis of the etiological profile of SCI in Brazil. Our findings suggest that the cervical region is the neurological level most affected (37.4%) and that motor vehicle accidents (37.3%) represent the most frequently observed cause in the pooled estimates, followed by falls (22.7%) and violence — including firearm injuries and physical assault (19.4%). This profile, characterized by the prominence of traffic-related injury alongside the substantial contribution of violence and the growing share of falls, can reflect the complex epidemiological landscape of Brazil, where persistent urban violence coexists with a progressive demographic transition.

However, these estimates must be interpreted with considerable caution. The high heterogeneity (I² 76%-95%) and the substantial risk of bias identified in the primary studies, compounded by evidence of publication bias for falls and violence, limit the generalizability of the results and suggest that the pooled proportions should be read as broad central tendencies rather than definitive national figures. The observed ranking of etiologies may be unstable and could reflect the composition of included studies rather than the true Brazilian SCI profile.

Therefore, there is an urgent need for methodologically robust, population-based research that is representative of all Brazilian regions. The findings underscore the necessity of improving the quality of epidemiological data collection and standardization of the scales used, an indispensable step to truly delineate the main etiologies in the country in a problem of such social impact, so that public policies can be implemented to promote more effective health care for this population.

## Supplementary Information

Supplementary Material 1

## Data Availability

The data that support the findings of this study are available from the primary studies cited in the article. Further details are available from the corresponding author upon reasonable request.
