# Supplementary material for: Epidemiological and etiological profile of spinal cord injury in Brazil: a systematic review and meta-analysis
Source: Inj Epidemiol. 2026 Sep 22;13:62. doi: 10.1186/s40621-026-00700-6 (PMC13595730; doi:10.1186/s40621-026-00700-6)
Supplement: Supplementary file 1 [file 40621_2026_700_MOESM1_ESM.docx]

**Supplemental Material**

**Search Strategies**

**Medline**

("Spinal Cord Injuries"[Mesh] OR "spinal cord injur*"[Title/Abstract] ) AND ( "epidemiology"[Subheading] OR "epidemiology"[Title/Abstract] OR "prevalence"[Title/Abstract] OR "incidence"[Title/Abstract] ) AND ( "Brazil"[Mesh] OR "Brazil"[Title/Abstract] OR "Brazilian"[Title/Abstract] )

This query resulted in 29 articles.

**LILACS**

(MH:"traumatismos da medula espinal" OR MH:"spinal cord injuries" OR TI:("lesão medular" OR "lesões medulares" OR "lesion medular" OR "lesiones medulares" OR "spinal cord injury" OR "spinal cord injuries") OR AB:("lesão medular" OR "lesões medulares" OR "lesion medular" OR "lesiones medulares" OR "spinal cord injury" OR "spinal cord injuries")) AND (MH:"epidemiologia" OR TI:(epidemiolog$ OR prevalênc$ OR incidênc$ OR epidemiolog$ OR prevalenc$ OR incidenc$ OR epidemiology OR prevalence OR incidence) OR AB:(epidemiolog$ OR prevalênc$ OR incidênc$ OR epidemiolog$ OR prevalenc$ OR incidenc$ OR epidemiology OR prevalence OR incidence)) AND (MH:"brasil" OR MH:"brazil" OR TI:(brasil OR brasileir$) OR AB:(brasil OR brasileir$))

This query resulted in 74 articles.

**Embase**

('spinal cord injury'/exp OR 'spinal cord injur*':ti,ab) AND ('epidemiology'/exp OR 'epidemiolog*':ti,ab OR 'prevalence':ti,ab OR 'incidence':ti,ab) AND ('brazil'/exp OR 'brazil':ti,ab OR 'brazilian':ti,ab) AND [embase]/lim AND [embase]/lim NOT [conference abstract]/lim

This query resulted in 60 articles.

**Sensitivity analysis**

**Table S1.** Sensitivity analysis restricted to confirmed spinal cord injury, compared with the primary analysis.

| **Outcome** | **Primary analysis** | | | | **Sensitivity analysis (restricted to confirmed SCI)** | | | |
| --- | --- | --- | --- | --- | --- | --- | --- | --- |
|  | **Studies (k)** | **Cases (N)** | **Proportion, % (95% CI)** | **I², %** | **Studies (k)** | **Cases (N)** | **Proportion, % (95% CI)** | **I², %** |
| ***Etiology of spinal cord injury*** | | | | | | | | |
| Motor vehicle accidents | 8 | 1109 | 37.3 (23.9–51.7) | 95 | 6 | 800 | 38.8 (29.9–48.0) | 84 |
| Falls | 8 | 1109 | 22.7 (16.2–29.9) | 85 | 6 | 800 | 19.1 (13.4–25.4) | 75 |
| Violence | 8 | 1109 | 19.4 (14.5–24.8) | 76 | 6 | 800 | 21.8 (17.8–26.2) | 44 |
| Shallow-water diving | 7 | 967 | 10.9 (5.1–18.3) | 89 | 5 | 658 | 13.7 (5.0–25.5) | 92 |
| ***Neurological level*** | | | | | | | | |
| Cervical injury | 6 | 1124 | 37.4 (27.0–48.4) | 93 | 5 | 874 | 40.2 (28.7–52.2) | 92 |

Pooled proportions were estimated with a random-effects model (DerSimonian–Laird) after the Freeman–Tukey double-arcsine transformation. The sensitivity analysis was restricted to studies whose numerator and denominator could be confined to patients with confirmed spinal cord injury (neurological deficit). For the etiology outcome, Castro et al. (39.0% Frankel E) and Souza Júnior et al. (26.8% Frankel E) were excluded because mechanism of injury was not reported separately for deficit-positive patients; for the neurological-level outcome, Souza Júnior et al. was excluded (Castro et al. did not contribute to the level pool). Shallow-water diving was reported as a distinct category by 7 studies in the primary analysis and 5 in the sensitivity analysis.

*CI*, confidence interval; *I²*, proportion of total variability attributable to between-study heterogeneity; *SCI*, spinal cord injury.

**Figure S1.** Pooled proportions of the four etiologic categories of spinal cord injury and of cervical injury, in the primary analysis and in the sensitivity analysis restricted to confirmed spinal cord injury.

Squares (primary) and diamonds (sensitivity) denote the random-effects pooled estimates; horizontal bars represent 95% confidence intervals. The sensitivity analysis excluded Castro and Souza Júnior (etiology) and Souza Júnior (neurological level), whose denominators included neurologically intact patients (Frankel E) that could not be separated by mechanism or level.
